# G Quadruplex in Plants: A Ubiquitous Regulatory Element and Its Biological Relevance

**DOI:** 10.3389/fpls.2017.01163

**Published:** 2017-07-04

**Authors:** Vikas Yadav, Hemansi, Nayun Kim, Narendra Tuteja, Puja Yadav

**Affiliations:** ^1^Department of Biochemistry, Central University of HaryanaMahendergarh, India; ^2^Department of Microbiology, Central University of HaryanaMahendergarh, India; ^3^Department of Microbiology and Molecular Genetics, University of Texas Health Science Center at Houston, HoustonTX, United States; ^4^The University of Texas Graduate School of Biomedical Sciences, HoustonTX, United States; ^5^Plant Molecular Biology Group, International Centre for Genetic Engineering and Biotechnology (ICGEB)New Delhi, India

**Keywords:** G quadruplex, genome stability, recombination, DNA damage and repair, transcriptional and translational regulation

## Abstract

G quadruplexes (G4) are higher-order DNA and RNA secondary structures formed by G-rich sequences that are built around tetrads of hydrogen-bonded guanine bases. Potential G4 quadruplex sequences have been identified in G-rich eukaryotic non-telomeric and telomeric genomic regions. Upon function, G4 formation is known to involve in chromatin remodeling, gene regulation and has been associated with genomic instability, genetic diseases and cancer progression. The natural role and biological validation of G4 structures is starting to be explored, and is of particular interest for the therapeutic interventions for human diseases. However, the existence and physiological role of G4 DNA and G4 RNA in plants species have not been much investigated yet and therefore, is of great interest for the development of improved crop varieties for sustainable agriculture. In this context, several recent studies suggests that these highly diverse G4 structures in plants can be employed to regulate expression of genes involved in several pathophysiological conditions including stress response to biotic and abiotic stresses as well as DNA damage. In the current review, we summarize the recent findings regarding the emerging functional significance of G4 structures in plants and discuss their potential value in the development of improved crop varieties.

## Introduction

**FIGURE 1:**
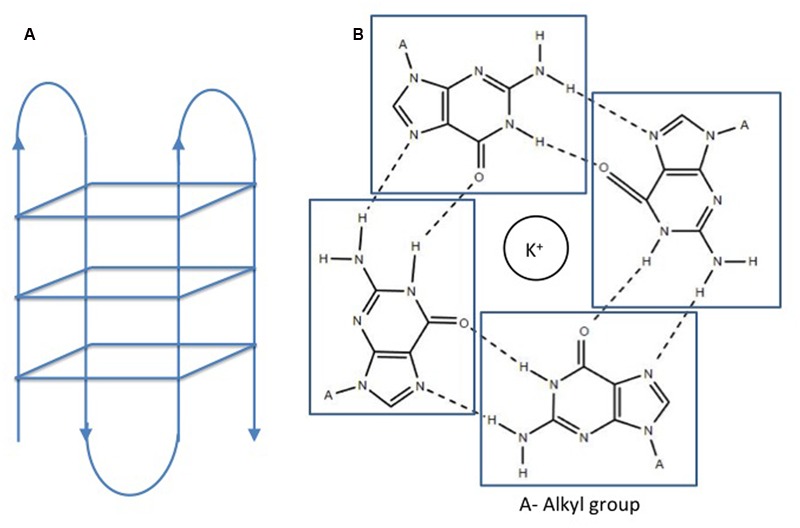
Structure of a G quadruplex. **(A)** Intramolecular G quadruplex. **(B)** G quartet formation in presence of cations (Huppert and Balasubramanian, 2005). No permission is required for the modification and reproduction of this figure under the terms of the Creative Commons CC BY license.

Double helical B-DNA is the predominant nucleic acid structure of the genome. In addition, DNA may adopt various extrahelical, non B-DNA secondary confirmations depending on the nucleotide content. These secondary structures are prevalent in all living organisms and play a pivotal role in the physiology of organisms. G quadruplex or G4 DNA is one of these structures adopted by spontaneous folding of sequences containing multiple runs of guanines (Bochman et al., 2012). Structurally, G4 DNA comprises of G-quartets or G-tetrads, in which the four guanine bases are bound together via Hoogsteen hydrogen bonds in a square planar conformation (Huppert and Balasubramanian, 2005). G-quartets stack on top of each other to form an advanced nucleic acid structure, G4 DNA (**Figure 1**). Adding to the complexity of the G4 DNA structure, the stacks of G-quartets are connected by loops of variable sizes (1–7 nucleotides) and orientations (parallel or antiparallel) (Wang and Patel, 1993; Parkinson et al., 2002; Huppert, 2010). These secondary structures are stabilized by cations, preferably potassium ion (K^+^) (Largy et al., 2016). G quadruplex structures form in RNA as well as DNA and may be intermolecular or intramolecular depending on number of nucleic acid strands involved in the quadruplex formation. For identification of potential G4 forming sequences in the in genome, G quadruplex prediction algorithms, such as Quadparser (Huppert and Balasubramanian, 2007), G4 calculator (Eddy and Maizels, 2006), and Quadbase (Dhapola and Chowdhury, 2016), are easily accessible and used widely. G quadruplex forming sequences (GQFS) have been categorized into different types on the basis of the number of guanine repeats (G2- two G4 repeats, G3- three G4 repeats, G4- four G4 repeats) and the number of nucleotide in the loop (1–3 bp, 1–7 bp., etc.) (**Table 1**). The stability of G4 structure depends on the length of loop with prediction of increased stability with shorter loop length. For instance, G3 type GQFS are more stable with 1–3 bp than 4–7 bp loop length, similarly G2 type GQFS are more stable with loop length of 1–2 bp than 3–4 bp (Bugaut and Balasubramanian, 2008).

**Table 1:** Different types of G quadruplexes forming sequences.

Types of GQFS	No. of guanine repeats	No. of nucleotides in loops	Sequence	Reference
G2 L1-2	2	1–2	GGNNGGNNGGNN	Bugaut and Balasubramanian, 2008
G2 L1-4	2	1–4	GGNNNNGGNNNNGG	Mullen et al., 2010
G2 L1-7	2	1–7	GGN_7_GGN_7_GGN_7_	Wang et al., 2015
G3 L1-3	3	1–3	GGGNNNGGGNNNGGG	Garg et al., 2016
G3 L1-7	3	1–7	GGGN_7_GGGN_7_GGGN_7_	Huppert and Balasubramanian, 2005

The study of G4 DNA has emerged as a forefront area of research because of its proposed role in several biological functions ranging from physiology to pathology. These secondary structures are found to be abundant in a wide range of eukaryotic and prokaryotic genomes. In bacteria, yeast and humans, genome-wide analyses of GQFS have revealed the non-random distribution of these secondary structures (König et al., 2010). It is evident that GQFS are particularly abundant in, but not limited to, promoters (Evans et al., 1984; Kilpatrick et al., 1986), telomeres **(**Blackburn, 1994**)**, ribosomal DNA (Sun et al., 1998), untranslated region (UTR) of mRNA, micro- and mini-satellite repeats (Nakagama et al., 2006), and immunoglobulin heavy chain switch regions (Yu et al., 2003).

Formation of these secondary structures has been associated with genomic instability and thus cancer progression. The highly significant correlation between GQFS and chromosomal translocation breakpoints in cancers demonstrates the biological relevance of G4-associated genome instability in mammalian cells. Studies in the model eukaryote *Saccharomyces cerevisiae* showed that topoisomerase enzymes and DNA helicases actively function to suppress G4-induced genome instability (Yadav et al., 2014, 2016).

In addition, occurence and distribution of these secondary structures in protooncogenes further suggest their role in the development and progression of cancer (Huppert and Balasubramanian, 2007; Verma et al., 2008). These protooncogenes includes *c-myc* (Siddiqui-Jain et al., 2002), *bcl-2* (Dexheimer et al., 2006), *c-kit* (Rankin et al., 2005; Fernando et al., 2006), *c-myb* (Palumbo et al., 2008), *KRAS* (Cogoi and Xodo, 2006; Cogoi et al., 2009) proto-oncogenes, *VEGF* (Sun et al., 2005), and *HIF-1* (De Armond et al., 2005).

In bacteria, GQFSs are evolutionarily conserved and enriched non-randomly in the promoter region of the genes that are associated with specific functions such as transcription, secondary metabolite biosynthesis, and signal transduction, suggesting a regulatory role of G4 DNA at global level in prokaryotes as well (Rawal et al., 2006; Beaume et al., 2013). In addition, G4 DNA and RNA play a key role in recombination-mediated antigenic variation mechanism that effectively varies the amino acid sequence of the surface expressed protein pilin and, thus, evades detection by the host adaptive immune system, in the bacterial pathogen *Neisseria gonorrhoeae* (Cahoon and Seifert, 2009).

The emerging pattern of association of GQFS with specific genomic regions suggests a regulatory role of GQFS in biologically significant pathways (Garg et al., 2016). However, in contrast to the significant information available on the distribution and role of G quadruplex-forming sequences in humans and microbial pathogens, similar studies in plant systems has been very limited. Recent investigation showed that G4 DNA and RNA are also generally conserved across plant species. In this context, several studies on bioinformatics analysis of plant genomes have been accompanied with identification and functional characterization of these secondary structures. In plants, genome wide distribution of G quadruplexes and their association with different genomic features led to the identification of putative G4 forming sequences within gene body or promoter region of orthologs genes in monocot and dicot plant species (Garg et al., 2016). Given the significant regulatory roles ascribed to G4 DNA in multiple systems, understanding the mechanism of gene regulation through G quadruplexes in plants may provide significant information for crop improvement. Very recently, several studies have been conducted to identify GQFS in a wide variety of plant species, including many important crop plants. Here, we will review the current state of understanding of the biological pathways where a significant role of G4 DNA is implicated.

## Distribution of G Quadruplex Forming Sequences in Plant Species

Similar to other organisms, the prevalence and distribution of GQFS in plant genomes vary according to the specific GQFS type. The G3 type GQFS were more abundant in the intergenic region, whereas, G2 type GQFS were found to be located in the genic region (**Table 1**). The specific association of different type of GQFS with different genomic regions suggest their vital role in various cellular processes for instance G2 GQFS may play a role in regulation of translation and transcription, while G3 GQFS are important for promoter regulation. In plant genome (*Arabidopsis thaliana, Oryza sativa, Glycine max, Cypripedium arietinum*), several G4 sequences were identified and confirmed to form parallel, antiparallel, intramolecular or intermolecular G4 DNA conformations *in vitro* by using circular dichroism (CD) spectroscopy and gel electrophoresis (Garg et al., 2016). Based on the gene ontology (GO) enrichment analyses, it have been shown that, in a variety of dicot plant species, orthologous genes harboring GQFS were involved in important biological pathways such as chromatin modification, regulating phosphorylation and intracellular signaling, auxin transport, seed development and GTPase activity. In monocot plant species, orthologous genes with GQFS are involved in biological processes such as development, ion transportation, regulation of transcription and protein folding (Garg et al., 2016).

## Evolutionary Conservation of G Quadruplex Among Plant Species

G4 DNA forming sequences are evolutionarily conserved from bacterial to single cell eukaryotes to metazoans. Among closely related fungal species, GQFS are evolutionary conserved at the nucleotide level and associated with distinct genomic features (Capra et al., 2010). In order to assess the evolutionary conservation of G4 sequences in plant species, a genome wide analysis was conducted for monocot and dicot plant genomes. The result conclusively showed that G2 type GQFS were abundant, comprising more than 90% of GQFS found in all the plant species analyzed, while G3 type GQFS were found less frequently, comprising 5% of the total GQFS in each of the plant species (Garg et al., 2016). In addition, frequency of GQFS distribution varied between monocot (∼80–1500 GQFS/Mb) and dicot (∼10–20 GQFS/Mb), this disparity in GQFS distribution may be due to high GC content of monocot genomes (Garg et al., 2016). The evolutionary conservation of GQFSs among plant species and their association with specific genomic features as described below suggest that G4 DNAs are integral parts of plant biology and are under evolutionary constraints.

## G Quadruplex Distribution and their Genomic Position: Functional Relevance

**FIGURE 2:**
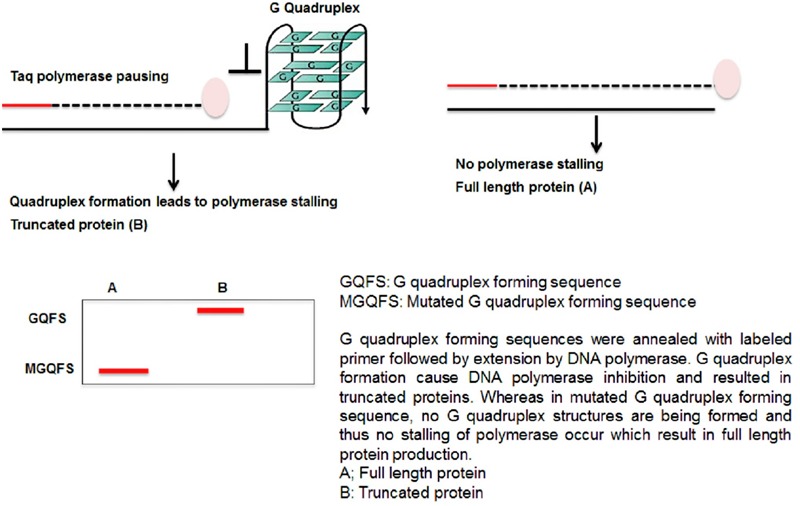
Genome wide occurance of G quadrplex forming sequences in different part of the gene (Garg et al., 2016).

G DNAs are considered a molecular switch for gene expression in metazoan cells (Eddy and Maizels, 2006), it is imperative to study the positional relationship of GQFS in plant genomes (**Figure 2**). In the genomes of *A. thaliana, Vitis vinifera, O. sativa* and *Populus trichocarpa*, GQFS are frequently located near the transcribed units or genes (Mullen et al., 2010). In particular, significant GQFS enrichment was observed in the transcription start site proximal regions [TPR], which are generally conserved across plant species. This suggests that G4 motifs in plants, similar to their proposed function in mammalian systems, play a role in regulating gene expression (Andorf et al., 2014). G4 motifs are also enriched at 5′ UTR, 3′ UTR, and 5′ end of introns implicating the role of G4 quadruplex in post-transcriptional regulation of the genes (Andorf et al., 2014; Wang et al., 2015). Comparative analyses of the genomes of *Oryza sativa japonica* and *O. sativa*, widely cultivated Asian variety of rice species, showed the enrichment of GQFS in TPR region (149.57GQFS/Mb and 131.34GQFS/Mb, respectively) relative to coding regions, introns, and 5′- and 3′-UTRs. Overall, the conserved pattern of high density of GQFS at TPR across the variety of plant species suggests the role of G quadruplex in transcriptional regulation in these species. Overall, the density of GQFS among monocot species was higher than that among dicot species studied (Wang et al., 2015).

G4 has also been identified in RNA in plants. For example, a combination of biophysical and biochemical assays was carried out to confirm G4 structure formation by a GQFS located in 5′ UTR of ATR mRNA in *A. thaliana* (Kwok et al., 2015). The ATR gene encodes a protein kinase, which is activated upon DNA damage and required for the ensuing DNA damage response of the cell including repair, cell cycle arrest and telomere maintenance. Further study into the role of the GQFS identified at the 5′ UT of the ATR mRNA showed its inhibitory effect during translation initiation. Search of ATR homologs among 31 plant species resulted in identification of 35 ATR homologs. At least one GQFS was present in the 5′ UTR of 16 (14 plant species) of 35 ATR mRNA. Whether the conserved GQFS present in the ATR mRNA also have conserved function such as the negative regulation of translation as in *A. thaliana* is yet to be resolved. GQFS mediated gene regulation appears to be prevalent and of functional importance in plant kingdom. **Table 2** describe the genome wide distribution of GQFS in different plant species.

**Table 2:** Properties of different types of G quadruplex in plant species.

	Type of	Genome wide	
Organism	GQFS	distribution	Reference
*Arabiodopsis thaliana*	G2 L1-4,	Higher in intergenice regions	Mullen et al., 2010
	G3 L1-3	Higher in genic regions	
*Vitis vinifera*	G3 L1-3	Higher in intergenice regions	Mullen et al., 2010
*Oryza sativa*	G2 L1-4	Higher in genic regions	Mullen et al., 2010
*Populus trichocarpa*	G3 L1-3	Higher in intergenice regions	Mullen et al., 2010
*Oryza japonicum*	G2 L1-7	Higher in intergenic regions	Wang et al., 2015
*Sapium sebiferum*	G3 L1-7	Higher in intergenic regions	Yang et al., 2015
*Zea mays*	G3 L1-7	Higher in genic regions	Andorf et al., 2014

Several putative G quadruplex structures have been identified by bioinformatics analyses in microRNA. In human genome, there are ∼16% of pre-mi-RNA that contains putative GQFS and can adopt these secondary structures to modulate canonical stem-loop structure of mi-RNA to adopt G quadruplex structure and thus impeded dicer mediated cleavage of mi-RNA (Mirihana Arachchilage et al., 2015). In addition, the equilibrium between the G quadruplex structure and stem loop structure influence the miRNA functionality as dicer enzyme recognize canonical stem loop structure in pre mi-RNA to produce mature miRNA and thus in turn formation of G quadruplex affect miRNA maturation [The RNA Stem–Loop to G Quadruplex Equilibrium Controls Mature MicroRNA Production inside the Cell (Pandey et al., 2015)]. *In silico* transcriptome wide analyses have identified significant number of G quadruplex motifs in human long non-coding RNA (lncRNA). Further, biophysical methods provide the information that approximately 60% of these putative structures form stable quadruplex *in vitro* and a further analyses of these secondary structures would give a better insight about the functional relevance of G4 structures in cellular function (Potential G quadruplexes in the human long non-coding transcriptome (Jayaraj et al., 2012). The plant genome have not been evaluated extensively for the presence of G quadruplex in their non-coding RNAs.

## G Quadruplex During Stress and DNA Damage: Biological Relevance

**FIGURE 3:**
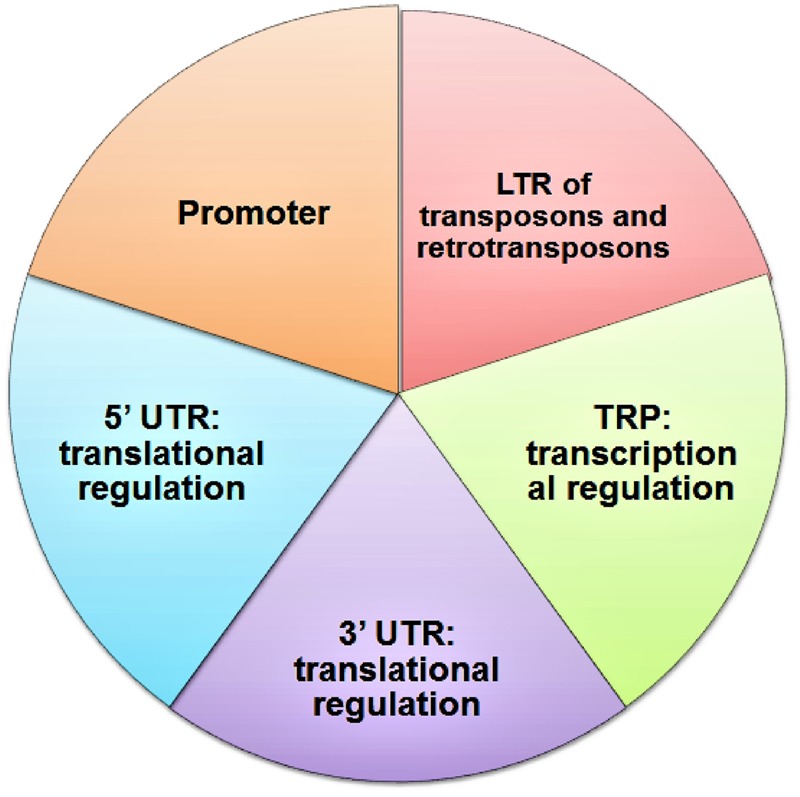
Putative role of G quadruplex in plants during physiological stress.

During unfavorable conditions such as abiotic (environmental factors such as high salt, high or low temperature) and biotic stress (damage to plants mediated by living organisms), plants must adapt to survive. During abiotic stress such as drought, the cytosolic concentrations of cations become elevated (Leigh and Wyn Jones, 1984). Since, higher potassium (K^+^) level is a condition that is known to facilitate the G quadruplex formation and under high salinity conditions, the K^+^ ion concentration in the cell increases (Zhang and Blumwald, 2001). This elevated levels of K^+^ ions facilitate the formation of G quadruplex genome wide and might be involved in salinity tolerance. Differential gene regulation mediated by G4 DNA or RNA structure formation is thereby hypothesized to be a potential mechanism to cope up with drought conditions. This hypothesis recently gained support by Mullen et al. (2010) who showed that GQFS are enriched at those genes differentially regulated during drought. In this study, transcriptome study of *Arabidopsis* originally conducted by Matsui et al. (2008) were analyzed to conclude that 16% of all genes in *A. thaliana* are drought-responsive and 45% of these genes contained at least one GQFS. Many similar studies followed since, (Andorf et al., 2014) demonstrated the abundance of GQFS in hypoxia-responsive genes in *maize* (Bailey-Serres et al., 2012). GQFS were also found to be frequent in genes associated with energy homeostasis signaling as well as many genes associated with TOR, AMP kinase, and oxidative stress signaling pathways. Kinases in TOR pathway are also directly regulated by the level of sugar availability and play a crucial role in nutrient and energy sensing. Occurrence of GQFS in genes encoding these kinases suggests that G quadruplex plays important role in regulation, signaling, and metabolic adjustment to energy status (Xu et al., 2010; Robaglia et al., 2012; Dobrenel et al., 2013; **Figure 3**). In *Sapium sebiferum* or Chinese tallow, which is an important agricultural crop species in east Asian countries, a bioinformatic analysis predicted the enrichment of GQFS at genes in the lipid biosynthesis and stress response pathways (Yang et al., 2015). Overall, genes that are differentially regulated during various stress conditions are more likely to contain a GQFS, and formation of G quadruplex may be one of multiple adaptive mechanisms utilized by plants during environmental stresses. Understanding how the stress response pathways are regulated in agriculturally important plant species through identification and functional analyses of GQFS can facilitate development of stress-tolerant plant varieties possibly through transgenic techniques and ultimately lead to higher-yield crops (Yang et al., 2015).

**FIGURE 4:**
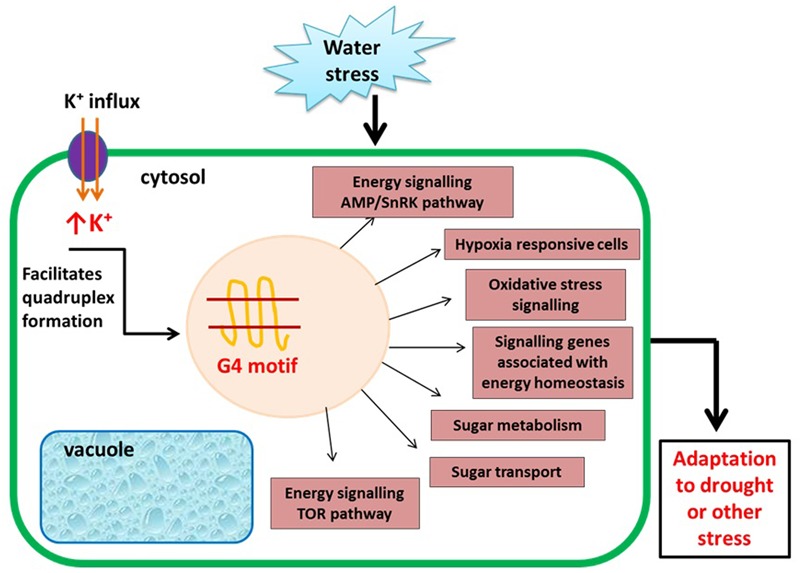
Impediment of DNA Polymerase activity by G quadruplex.

G4-forming synthetic oligonucleotides impede DNA polymerase activity *in vitro* and produced truncated product in presence of K^+^ ions, which stabilizes G4 structure (Garg et al., 2016). This result leads to the postulation that replication would be obstructed by G4 DNA *in vivo*, causing replication fork stall and collapse and thus causes genome instability (**Figure 4**).

## Transposable Elements and G Quadruplex Formation

Transposable elements are a significant part of eukaryotic genomes as they contain many regulatory sequences and serve as machinery to disseminate the genes present within. Interestingly, GQFS has been found to be present in long terminal repeats (LTR) of plant-transposons and -retrotransposons and possibly effects not only transcription and translation but reverse transcription as well. (Lexa et al., 2014; Kejnovsky et al., 2015). Formation of these secondary structures causes the conformational changes in DNA and DNA can become nucleosome free when its confirmation changes and It is well known that preferred site of integration for TE is open chromatin (Huppert and Balasubramanian, 2005; Liu et al., 2009; Wong and Huppert, 2009). G quadruplex in TE inhibits transcription and formation of these structures in TE can serve as hot spot for recombination and TE serves a vehicle for spread of G4 structure in the genome. These secondary structure are not only formed inside the TE due to open chromatin confirmation but can also become the genomic targets for insertion of new TE during which changed DNA confirmations are recognized by transposase or by integrase. Proteins originating from TE retain their affinity to open configuration and prefer binding to secondary structures such as G4 DNA, i.e., RAG1 protein (Nambiar and Raghavan, 2011). Formation of G quadruplex structures in TE-derived RNA participate in many important cellular processes (Kapusta et al., 2013).

Moreover, long stretches of guanines were identified upstream and downstream of the promoter region of retrotransposons. These sequences have been confirmed to readily adopt parallel- or antiparallel-stranded G quadruplexes by CD spectroscopy (Lexa et al., 2014). Occurrence of GQFS, at these specific locations suggests their role during initiation of transcription and elongation of retrotransposon RNA. Alternatively, these GQFS might act as the check point of transcription and reverse transcription. Biological role of GQFS in transposable elements life cycle have been established, as long stretch of guanines is found in young and active LTR and lower number of guanines is found in old elements due to supress elongation of RNA strand (Lexa et al., 2014). Such enrichment of GQFS, within specific region of LTR retrotransposons propose functional role of G quadruplex in genome instability (recombination based reshuffling) of plant genomes (Lexa et al., 2014; Kejnovsky et al., 2015).

## G Quadruplex Binding Proteins in Plants

Several G quadruplex binding proteins have been identified in yeast and metazoans. Upon binding, these proteins may either help resolve the G4 structures or may enhance the stability of the secondary structures. G4 resolving proteins include DNA helicases such as BLM (RecQ family), WRN (RecQ family), and FANCJ in humans (Sun et al., 1998; Fry and Loeb, 1999; Mohaghegh et al., 2001; Cheok et al., 2005; Wu et al., 2008), Dog-1 in the nematode *Caenorhabditis elegans* (Youds et al., 2008), and Sgs1 (RecQ family) and Pif1 in yeast (Eddy and Maizels, 2006).

RecQ helicases, conserved from bacteria to humans, are involved in unwinding of a wide variety of DNA substrates including G quadruplexes and thus are important in maintaining genome integrity. In humans, there are five RecQ-family helicases (BLM, WRN, RTS/RECQ4) while yeast and bacteria possess only one – Sgs1 and RecQ, respectively. In case of plants, *A. thaliana* genome contains seven different genes that encode RecQ family helicase; RECQ1, RECQ2, RECQ3, RECQ4, RECQ4B, RECQ5, and RECQsim (Hartung et al., 2000; Hartung and Puchta, 2006). *At*RecQ4A and 4B are two of the genes of this family that evolved due to recent duplication and are 70% identical on protein level to other members of this family. Role of *At*RecQ4A have been suggested to be equivalent to yeast Sgs1 and human BLM. *At*RecQ4B is distinct among all eukaryotic RecQ homologs as it appears to promote rather than suppress crossover recombinations (Hartung et al., 2007; Schröpfer et al., 2014). RecQsim contains a unique insertion of acidic amino acids in its helicase domain. Homologs of RecQsim of *A. thaliana* have been found in other plant species including rice and rape (Bagherieh-Najjar et al., 2003). The expression of *A. thaliana* RecQsim gene in yeast lacking Sgs1 compensates the loss of Sgs1 and rescues the hypersenstivity to the DNA damaging drug methyl methanesulfonate (MMS), indicating functional conservation between these helicases.

In yeast, other non-helicase G4 DNA binding proteins, such as the co-transcription factor Sub1, also contribute to the stability of the genomic loci containing GQFS (Lopez et al., 2017). In case of plants, very little information is available regarding potential G4-binding proteins. In maize (*Zea mays*), a G4 binding protein known as *ZmNDPK1* have been identified in a a ligand-binding screening of a cDNA-expression library. *ZmND*PK1, which is a nucleoside diphosphate kinase 1, interacts with folded GQFS-containing oligos, with low nanomolar-range affinity (Kopylov et al., 2015). Electrophoretic mobility shift assay (EMSA) using nuclear extracts from rice plants revealed proteins stably binding G4 DNA of both parallel and antiparallel conformations; the identities of these proteins, however, are still pending (Garg et al., 2016). Additionally, certain medicinal plant extracts such as theaflavin-digallate from tea and saffron carotenoids from *Crocus sativus*, were shown to contain non-protein, small molecule ligands with G quadruplex binding activity (Hoshyar et al., 2012; Mikutis et al., 2013; Wang et al., 2015). As shown in yeast, the interaction of GQFS with proteins with specific affinity to the non-canonical secondary structure is an important mechanism in G4-associated regulatory functions. Therefore, in order to fully understand the function of G4 in plant biology, identification and further characterization of G4 binding proteins and small molecule G4 ligands in plant species are of high priority.

## Conclusions and Perspectives

Genome-wide analyses elucidated numerous GQFS in several plant species including *A. thaliana, Z. mays, O. japonicum* and *O. sativa* and others and conclusively showed the abundance of G2 type GQFS in the genic and coding regions and G3 type GQFS in the intergenic regions. Biophysical characterization of a subset of these GQFSs have also been accomplished. G quadruplex play a regulatory role during cellular responses to DNA damage and other internal and external cues such as sugar availability; metabolic and energy status; stress response etcs. In addition, G quadruplex formation can induce fluorescence activation with high selectivity and sensitivity (DasGupta et al., 2015). Current agricultural production is heavily reliant on many biotic and abiotic factors; stress conditions such as drought and soil salinity are main factors responsible for crop yield reduction. Further work into Identification and functional analyses of G quadruplexes in plants can be a particular interest as a target for biotic and abiotic stress response in plants. It is not yet clear whether the tolerance mechanisms of plants in response to various kind of stresses are directly or indirectly regulated by G4 quadruplexes. Future work is expected to focus on defining the detailed the molecular pathway(s) governed by G4 DNA in response to both biotic and abiotic stresses. Generating transgenic plants tolerant to drought and other stresses is a central hypothesis for the agricultural industry. Understanding how the structural transformation of G4 DNA assembly is regulated and in turn regulate gene expression, therefore, could have valuable implications for the development of transgenic plant varieties with higher yield.

## Author Contributions

PY and VY contributed to the conception of the review article. PY and VY drafted the work. PY, VY, H, and NK wrote the review article. PY, VY, NT, and NK revised it critically. PY, NK, and NT helped in literature search. PY, VY, H, NT, and NK gave Final approval of the version to be published.

## Conflict of Interest Statement

The authors declare that the research was conducted in the absence of any commercial or financial relationships that could be construed as a potential conflict of interest.

## References

[B1] Andorf C. M. Kopylov M. Dobbs D. Koch K. E. Stroupe M. E. Lawrence C. J. (2014). G-quadruplex (G4) motifs in the maize (*Zea mays* L.) genome are enriched at specific locations in thousands of genes coupled to energy status, hypoxia, low sugar, and nutrient deprivation. *J. Genet. Genomics* 41 627–647. 10.1016/j.jgg.2014.10.004 25527104

[B2] Bagherieh-Najjar M. B. de Vries O. M. H. Kroon J. T. M. Wright E. L. Elborough K. M. Hille J. (2003). Arabidopsis RecQsim, a plant-specific member of the RecQ helicase family, can suppress the MMS hypersensitivity of the yeast sgs1 mutant. *Plant Mol. Biol.* 52 273–284. 12856935 10.1023/a:1023968429220

[B3] Bailey-Serres J. Fukao T. Gibbs D. J. Holdsworth M. J. Lee S. C. Licausi F. (2012). Making sense of low oxygen sensing. *Trends Plant Sci.* 17 129–138. 10.1016/j.tplants.2011.12.004 22280796

[B4] Beaume N. Pathak R. Yadav V. K. Kota S. Misra H. S. Gautam H. K. (2013). Genome-wide study predicts promoter-G4 DNA motifs regulate selective functions in bacteria: radioresistance of *D. radiodurans* involves G4 DNA-mediated regulation. *Nucleic Acids Res.* 41 76–89. 10.1093/nar/gks1071 23161683 PMC3592403

[B5] Blackburn E. H. (1994). Telomeres: no end in sight. *Cell* 77 621–623. 10.1016/0092-8674(94)90046-9 8205611

[B6] Bochman M. L. Paeschke K. Zakian V. A. (2012). DNA secondary structures: stability and function of G-quadruplex structures. *Nat. Rev. Genet.* 13 770–780. 10.1038/nrg3296 23032257 PMC3725559

[B7] Bugaut A. Balasubramanian S. (2008). A sequence-independent study of the influence of short loop lengths on the stability and topology of intramolecular DNA G-quadruplexes. *Biochemistry* 47 689–697. 10.1021/bi701873c 18092816 PMC2408741

[B8] Cahoon L. A. Seifert H. S. (2009). An alternative DNA structure is necessary for pilin antigenic variation in *Neisseria gonorrhoeae*. *Science* 325 764–767. 10.1126/science.1175653 19661435 PMC2803317

[B9] Capra J. A. Paeschke K. Singh M. Zakian V. A. (2010). G-quadruplex DNA sequences are evolutionarily conserved and associated with distinct genomic features in *Saccharomyces cerevisiae*. *PLoS Comput. Biol.* 6:e1000861 10.1371/journal.pcbi.1000861 PMC2908698 20676380

[B10] Cheok C. F. Bachrati C. Z. Chan K. L. Ralf C. Wu L. Hickson I. D. (2005). Roles of the Bloom’s syndrome helicase in the maintenance of genome stability. *Biochem. Soc. Trans.* 33 1456–1459. 10.1042/BST0331456 16246145

[B11] Cogoi S. Paramasivam M. Filichev V. Géci I. Pedersen E. B. Xodo L. E. (2009). Identification of a new G-quadruplex motif in the KRAS promoter and design of pyrene-modified G4-decoys with antiproliferative activity in pancreatic cancer cells. *J. Med. Chem.* 52 564–568. 10.1021/jm800874t 19099510

[B12] Cogoi S. Xodo L. E. (2006). G-quadruplex formation within the promoter of the KRAS proto-oncogene and its effect on transcription. *Nucleic Acids Res.* 34 2536–2549. 10.1093/nar/gkl286 16687659 PMC1459413

[B13] DasGupta S. Shelke S. A. Li N. Piccirilli J. A. (2015). Spinach RNA aptamer detects lead(II) with high selectivity. *Chem. Commun.* 51 9034–9037. 10.1039/c5cc01526j PMC4755583 25940073

[B14] De Armond R. Wood S. Sun D. Hurley L. H. Ebbinghaus S. W. (2005). Evidence for the presence of a guanine quadruplex forming region within a polypurine tract of the hypoxia inducible factor 1alpha promoter. *Biochemistry* 44 16341–16350. 10.1021/bi051618u 16331995

[B15] Dexheimer T. S. Sun D. Hurley L. H. (2006). Deconvoluting the structural and drug-recognition complexity of the G-quadruplex-forming region upstream of the bcl-2 P1 promoter. *J. Am. Chem. Soc.* 128 5404–5415. 10.1021/ja0563861 16620112 PMC2580050

[B16] Dhapola P. Chowdhury S. (2016). QuadBase2: web server for multiplexed guanine quadruplex mining and visualization. *Nucleic Acids Res.* 44 W277–W283. 10.1093/nar/gkw425 27185890 PMC4987949

[B17] Dobrenel T. Marchive C. Azzopardi M. Clément G. Moreau M. Sormani R. (2013). Sugar metabolism and the plant target of rapamycin kinase: a sweet operaTOR? *Front. Plant Sci.* 4:93 10.3389/fpls.2013.00093 PMC3640205 23641244

[B18] Eddy J. Maizels N. (2006). Gene function correlates with potential for G4 DNA formation in the human genome. *Nucleic Acids Res.* 34 3887–3896. 10.1093/nar/gkl529 16914419 PMC1557811

[B19] Evans T. Schon E. Gora-Maslak G. Patterson J. Efstratiadis A. (1984). S1-hypersensitive sites in eukaryotic promoter regions. *Nucleic Acids Res.* 12 8043–8058. 10.1093/nar/12.21.8043 6095186 PMC320272

[B20] Fernando H. Reszka A. P. Huppert J. Ladame S. Rankin S. Venkitaraman A. R. (2006). A conserved quadruplex motif located in a transcription activation site of the human c-kit oncogene. *Biochemistry* 45 7854–7860. 10.1021/bi0601510 16784237 PMC2195898

[B21] Fry M. Loeb L. A. (1999). Human werner syndrome DNA helicase unwinds tetrahelical structures of the fragile X syndrome repeat sequence d(CGG)n. *J. Biol. Chem.* 274 12797–12802. 10.1074/jbc.274.18.12797 10212265

[B22] Garg R. Aggarwal J. Thakkar B. (2016). Genome-wide discovery of G-quadruplex forming sequences and their functional relevance in plants. *Sci. Rep.* 6:28211 10.1038/srep28211 PMC4914980 27324275

[B23] Hartung F. Plchová H. Puchta H. (2000). Molecular characterisation of RecQ homologues in *Arabidopsis thaliana*. *Nucleic Acids Res.* 28 4275–4282. 11058127 10.1093/nar/28.21.4275 PMC113147

[B24] Hartung F. Puchta H. (2006). The RecQ gene family in plants. *J. Plant Physiol.* 163 287–296. 10.1016/j.jplph.2005.10.013 16371241

[B25] Hartung F. Suer S. Puchta H. (2007). Two closely related RecQ helicases have antagonistic roles in homologous recombination and DNA repair in *Arabidopsis thaliana*. *Proc. Natl. Acad. Sci. U.S.A.* 104 18836–18841. 10.1073/pnas.0705998104 18000056 PMC2141863

[B26] Hoshyar R. Bathaie S. Z. Kyani A. Mousavi M. F. (2012). Is there any interaction between telomeric DNA structures, G-quadruplex and I-motif, with saffron active metabolites? *Nucleosides Nucleotides Nucleic Acids* 31 801–812. 10.1080/15257770.2012.730164 23145950

[B27] Huppert J. L. (2010). Structure, location and interactions of G-quadruplexes. *FEBS J.* 277 3452–3458. 10.1111/j.1742-4658.2010.07758.x 20670279

[B28] Huppert J. L. Balasubramanian S. (2005). Prevalence of quadruplexes in the human genome. *Nucleic Acids Res.* 33 2908–2916. 10.1093/nar/gki609 15914667 PMC1140081

[B29] Huppert J. L. Balasubramanian S. (2007). G-quadruplexes in promoters throughout the human genome. *Nucleic Acids Res.* 35 406–413. 10.1093/nar/gkl1057 17169996 PMC1802602

[B30] Jayaraj G. G. Pandey S. Scaria V. Maiti S. (2012). Potential G-quadruplexes in the human long non-coding transcriptome. *RNA Biol.* 9 81–86. 10.4161/rna.9.1.18047 22258148

[B31] Kapusta A. Kronenberg Z. Lynch V. J. Zhuo X. Ramsay L. Bourque G. (2013). Transposable elements are major contributors to the origin, diversification, and regulation of vertebrate long noncoding RNAs. *PLoS Genet.* 9:e1003470 10.1371/journal.pgen.1003470 PMC3636048 23637635

[B32] Kejnovsky E. Tokan V. Lexa M. (2015). Transposable elements and G-quadruplexes. *Chromosome Res.* 23 615–623. 10.1007/s10577-015-9491-7 26403244

[B33] Kilpatrick M. W. Torri A. Kang D. S. Engler J. A. Wells R. D. (1986). Unusual DNA structures in the adenovirus genome. *J. Biol. Chem.* 261 11350–11354. 3015967

[B34] König S. L. B. Evans A. C. Huppert J. L. (2010). Seven essential questions on G-quadruplexes. *Biomol. Concepts* 1 197–213. 10.1515/bmc.2010.011 25961997

[B35] Kopylov M. Bass H. W. Stroupe M. E. (2015). The maize (*Zea mays* L.) nucleoside diphosphate kinase1 (ZmNDPK1) gene encodes a human NM23-H2 homologue that binds and stabilizes G-quadruplex DNA. *Biochemistry* 54 1743–1757. 10.1021/bi501284g 25679041

[B36] Kwok C. K. Ding Y. Shahid S. Assmann S. M. Bevilacqua P. C. (2015). A stable RNA G-quadruplex within the 5’-UTR of *Arabidopsis thaliana ATR* mRNA inhibits translation. *Biochem. J.* 467 91–102. 10.1042/BJ20141063 25793418

[B37] Largy E. Mergny J. -L. Gabelica V. (2016). Role of alkali metal ions in G-quadruplex nucleic acid structure and stability. *Met. Ions Life Sci.* 16 203–258. 10.1007/978-3-319-21756-7_7 26860303

[B38] Leigh R. A. Wyn Jones R. G. (1984). A hypothesis relating critical potassium concentrations for growth to the distribution and functions of this ion in the plant cell. *New Phytol.* 97 1–13. 10.1111/j.1469-8137.1984.tb04103.x

[B39] Lexa M. Kejnovsky E. Steflova P. Konvalinova H. Vorlickova M. Vyskot B. (2014). Quadruplex-forming sequences occupy discrete regions inside plant LTR retrotransposons. *Nucleic Acids Res.* 42 968–978. 10.1093/nar/gkt893 24106085 PMC3902901

[B40] Liu S. Yeh C. -T. Ji T. Ying K. Wu H. Tang H. M. (2009). Mu transposon insertion sites and meiotic recombination events co-localize with epigenetic marks for open chromatin across the maize genome. *PLoS Genet.* 5:e1000733 10.1371/journal.pgen.1000733 PMC2774946 19936291

[B41] Lopez C. R. Singh S. Hambarde S. Griffin W. C. Gao J. Chib S. (2017). Yeast Sub1 and human PC4 are G-quadruplex binding proteins that suppress genome instability at co-transcriptionally formed G4 DNA. *Nucleic Acids Res.* 45 5850–5862. 10.1093/nar/gkx201 28369605 PMC5449603

[B42] Matsui A. Ishida J. Morosawa T. Mochizuki Y. Kaminuma E. Endo T. A. (2008). *Arabidopsis* transcriptome analysis under drought, cold, high-salinity and ABA treatment conditions using a tiling array. *Plant Cell Physiol.* 49 1135–1149. 10.1093/pcp/pcn101 18625610

[B43] Mikutis G. Karaköse H. Jaiswal R. LeGresley A. Islam T. Fernandez-Lahore M. (2013). Phenolic promiscuity in the cell nucleus–epigallocatechingallate (EGCG) and theaflavin-3,3’-digallate from green and black tea bind to model cell nuclear structures including histone proteins, double stranded DNA and telomeric quadruplex DNA. *Food Funct.* 4 328–337. 10.1039/c2fo30159h 23172122

[B44] Mirihana Arachchilage G. Dassanayake A. C. Basu S. (2015). A potassium ion-dependent RNA structural switch regulates human pre-miRNA 92b maturation. *Chem. Biol.* 22 262–272. 10.1016/j.chembiol.2014.12.013 25641166

[B45] Mohaghegh P. Karow J. K. Brosh R. M. Jr. Bohr V. A. Hickson I. D. (2001). The Bloom’s and Werner’s syndrome proteins are DNA structure-specific helicases. *Nucleic Acids Res.* 29 2843–2849. 10.1093/nar/29.13.2843 11433031 PMC55766

[B46] Mullen M. A. Olson K. J. Dallaire P. Major F. Assmann S. M. Bevilacqua P. C. (2010). RNA G-Quadruplexes in the model plant species *Arabidopsis thaliana*: prevalence and possible functional roles. *Nucleic Acids Res.* 38 8149–8163. 10.1093/nar/gkq804 20860998 PMC3001093

[B47] Nakagama H. Higuchi K. Tanaka E. Tsuchiya N. Nakashima K. Katahira M. (2006). Molecular mechanisms for maintenance of G-rich short tandem repeats capable of adopting G4 DNA structures. *Mutat. Res.* 598 120–131. 10.1016/j.mrfmmm.2006.01.014 16513142

[B48] Nambiar M. Raghavan S. C. (2011). How does DNA break during chromosomal translocations? *Nucleic Acids Res.* 39 5813–5825. 10.1093/nar/gkr223 21498543 PMC3152359

[B49] Palumbo S. L. Memmott R. M. Uribe D. J. Krotova-Khan Y. Hurley L. H. Ebbinghaus S. W. (2008). A novel G-quadruplex-forming GGA repeat region in the c-myb promoter is a critical regulator of promoter activity. *Nucleic Acids Res.* 36 1755–1769. 10.1093/nar/gkm1069 18252774 PMC2330228

[B50] Pandey S. Agarwala P. Jayaraj G. G. Gargallo R. Maiti S. (2015). The RNA stem–loop to G-quadruplex equilibrium controls mature MicroRNA production inside the cell. *Biochemistry* 54 7067–7078. 10.1021/acs.biochem.5b00574 26554903

[B51] Parkinson G. N. Lee M. P. H. Neidle S. (2002). Crystal structure of parallel quadruplexes from human telomeric DNA. *Nature* 417 876–880. 10.1038/nature755 12050675

[B52] Rankin W. W. Brennan S. Schell E. Laviwa J. Rankin S. H. (2005). The stigma of being HIV-positive in Africa. *PLoS Med.* 2:e247 10.1371/journal.pmed.0020247 PMC1176240 16008508

[B53] Rawal P. Kummarasetti V. B. R. Ravindran J. Kumar N. Halder K. Sharma R. (2006). Genome-wide prediction of G4 DNA as regulatory motifs: role in *Escherichia coli* global regulation. *Genome Res.* 16 644–655. 10.1101/gr.4508806 16651665 PMC1457047

[B54] Robaglia C. Thomas M. Meyer C. (2012). Sensing nutrient and energy status by SnRK1 and TOR kinases. *Curr. Opin. Plant Biol.* 15 301–307. 10.1016/j.pbi.2012.01.012 22305521

[B55] Schröpfer S. Kobbe D. Hartung F. Knoll A. Puchta H. (2014). Defining the roles of the N-terminal region and the helicase activity of RECQ4A in DNA repair and homologous recombination in *Arabidopsis*. *Nucleic Acids Res.* 42 1684–1697. 10.1093/nar/gkt1004 24174542 PMC3919593

[B56] Siddiqui-Jain A. Grand C. L. Bearss D. J. Hurley L. H. (2002). Direct evidence for a G-quadruplex in a promoter region and its targeting with a small molecule to repress c-MYC transcription. *Proc. Natl. Acad. Sci. U.S.A.* 99 11593–11598. 10.1073/pnas.182256799 12195017 PMC129314

[B57] Sun D. Guo K. Rusche J. J. Hurley L. H. (2005). Facilitation of a structural transition in the polypurine/polypyrimidine tract within the proximal promoter region of the human VEGF gene by the presence of potassium and G-quadruplex-interactive agents. *Nucleic Acids Res.* 33 6070–6080. 10.1093/nar/gki917 16239639 PMC1266068

[B58] Sun H. Karow J. K. Hickson I. D. Maizels N. (1998). The Bloom’s syndrome helicase unwinds G4 DNA. *J. Biol. Chem.* 273 27587–27592. 10.1074/jbc.273.42.27587 9765292

[B59] Verma A. Halder K. Halder R. Yadav V. K. Rawal P. Thakur R. K. (2008). Genome-wide computational and expression analyses reveal G-quadruplex DNA motifs as conserved cis-regulatory elements in human and related species. *J. Med. Chem.* 51 5641–5649. 10.1021/jm800448a 18767830

[B60] Wang Y. Patel D. J. (1993). Solution structure of a parallel-stranded G-quadruplex DNA. *J. Mol. Biol.* 234 1171–1183. 10.1006/jmbi.1993.1668 8263919

[B61] Wang Y. Zhao M. Zhang Q. Zhu G.-F. Li F.-F. Du L.-F. (2015). Genomic distribution and possible functional roles of putative G-quadruplex motifs in two subspecies of *Oryza sativa*. *Comput. Biol. Chem.* 56 122–130. 10.1016/j.compbiolchem.2015.04.009 25935116

[B62] Wong H. M. Huppert J. L. (2009). Stable G-quadruplexes are found outside nucleosome-bound regions. *Mol. Biosyst.* 5 1713–1719. 10.1039/b905848f 19585004

[B63] Wu Y. Shin-ya K. Brosh R. M. (2008). FANCJ helicase defective in Fanconia anemia and breast cancer unwinds G-quadruplex DNA to defend genomic stability. *Mol. Cell. Biol.* 28 4116–4128. 10.1128/MCB.02210-07 18426915 PMC2423121

[B64] Xu X. M. Lin H. Maple J. Björkblom B. Alves G. Larsen J. P. (2010). The *Arabidopsis* DJ-1a protein confers stress protection through cytosolic SOD activation. *J. Cell Sci.* 123 1644–1651. 10.1242/jcs.063222 20406884

[B65] Yadav P. Harcy V. Argueso J. L. Dominska M. Jinks-Robertson S. Kim N. (2014). Topoisomerase I plays a critical role in suppressing genome instability at a highly transcribed G-quadruplex-forming sequence. *PLoS Genet.* 10:e1004839 10.1371/journal.pgen.1004839 PMC4256205 25473964

[B66] Yadav P. Owiti N. Kim N. (2016). The role of topoisomerase I in suppressing genome instability associated with a highly transcribed guanine-rich sequence is not restricted to preventing RNA:DNA hybrid accumulation. *Nucleic Acids Res.* 44 718–729. 10.1093/nar/gkv1152 26527723 PMC4737143

[B67] Yang M. Wu Y. Jin S. Hou J. Mao Y. Liu W. (2015). Flower bud transcriptome analysis of *Sapium sebiferum* (Linn.) Roxb. and primary investigation of drought induced flowering: pathway construction and G-quadruplex prediction based on transcriptome. *PLoS ONE* 10:e0118479 10.1371/journal.pone.0118479 PMC4349590 25738565

[B68] Youds J. L. Barber L. J. Ward J. D. Collis S. J. O’Neil N. J. Boulton S. J. (2008). DOG-1 is the *Caenorhabditis elegans* BRIP1/FANCJ homologue and functions in interstrand cross-link repair. *Mol. Cell. Biol.* 28 1470–1479. 10.1128/MCB.01641-07 18086896 PMC2258786

[B69] Yu K. Chedin F. Hsieh C.-L. Wilson T. E. Lieber M. R. (2003). R-loops at immunoglobulin class switch regions in the chromosomes of stimulated B cells. *Nat. Immunol.* 4 442–451. 10.1038/ni919 12679812

[B70] Zhang H. X. Blumwald E. (2001). Transgenic salt-tolerant tomato plants accumulate salt in foliage but not in fruit. *Nat. Biotechnol.* 19 765–768. 10.1038/90824 11479571

